# Intrafractional Motion in Online-Adaptive Magnetic Resonance-Guided Radiotherapy of Adrenal Metastases Leads to Reduced Target Volume Coverage and Elevated Organ-at-Risk Doses

**DOI:** 10.3390/cancers17091533

**Published:** 2025-04-30

**Authors:** Philipp Hoegen-Saßmannshausen, Tobias P. Hartschuh, Claudia Katharina Renkamp, Carolin Buchele, Fabian Schlüter, Elisabetta Sandrini, Fabian Weykamp, Sebastian Regnery, Eva Meixner, Laila König, Jürgen Debus, Sebastian Klüter, Juliane Hörner-Rieber

**Affiliations:** 1 Department of Radiation Oncology, Heidelberg University Hospital, 69210 Heidelberg, Germany; 2 Heidelberg Institute of Radiation Oncology (HIRO), 69120 Heidelberg, Germany; 3 National Center for Tumor Diseases (NCT), 69210 Heidelberg, Germany; 4 Clinical Cooperation Unit Radiation Oncology, German Cancer Research Center (DKFZ), 69210 Heidelberg, Germany; 5 Department of Radiation Oncology, RKH Klinikum Ludwigsburg, 71640 Ludwigsburg, Germany; 6 Department of Radiation Oncology, Heidelberg Ion Beam Therapy Center (HIT), Heidelberg University Hospital, 69210 Heidelberg, Germany; 7 German Cancer Consortium (DKTK), Partner Site Heidelberg, 69120 Heidelberg, Germany; 8 Department of Radiation Oncology, Düsseldorf University Hospital, 40225 Düsseldorf, Germany

**Keywords:** intrafractional changes, drift, peristalsis, gating, SBRT, SABR, MR-linac, oligometastasis

## Abstract

Background/Objectives: Stereotactic body radiotherapy is frequently used in patients with adrenal metastases. Motion of adherent radiosensitive organs at risk (OARs) and tumors influence OAR toxicity and tumor control. Online-adaptive Magnetic Resonance-guided radiotherapy (MRgRT) can address and mitigate interfractional changes. However, the impact of intrafractional variations in adrenal MRgRT is unknown. Methods: A total of 23 patients with 24 adrenal metastases were treated with MRgRT. After daily plan adaptation and before beam application, an additional (preRT) 3d MRI was acquired. PreRT target volumes and OARs were retrospectively recontoured in 200 fractions. The delivered, online-adapted treatment plans, as well as non-adapted baseline plans, were calculated on these re-contoured structures to quantify the dosimetric impact of intrafractional variations on target volume coverage and OAR doses with and without online adaptation. Normal tissue complication probabilities (NTCPs) were calculated. Results: The median time between the two MRIs was 56.4 min. GTV and PTV coverage (dose to 95% of the PTV, D95%, and volume covered by 100% of the prescription dose, V100%) were significantly inferior in the preRT plans. GTV D_mean_ was significantly impaired in left-sided metastases, but not in right-sided metastases. Compared to non-adapted preRT plans, adapted preRT plans were still significantly superior for all GTV and PTV metrics. Intrafractional violations of OAR constraints were frequent. D0.5cc and the volume exposed to the near-maximum dose constraint were significantly higher in the preRT plans. The volume exposed to the D0.5cc constraints in single fractions escalated up to 1.5 cc for the esophagus, 3.2 cc for the stomach, 5.3 cc for the duodenum and 7.3 cc for the bowel. This led to significantly elevated NTCPs for the stomach, bowel and duodenum. Neither PTV D95%, nor gastrointestinal OAR maximum doses were significantly impaired by longer fraction duration. Conclusions: Intrafractional motion in adrenal MRgRT caused significant impairment of target volume coverage (D95% and V100%), potentially undermining local control. Frequent violation of gastrointestinal OAR constraints led to elevated NTCP. Compared to non-adaptive treatment, online adaptation still highly improved GTV and PTV coverage.

## Simple Summary

Adrenal metastases can be precisely treated with online-adaptive MR-guided radiotherapy accounting for tumor and organ-at-risk (OAR) motion and variation between treatment sessions. However, changes also occur during treatment due to breathing, patient and organ motion. The impact of these changes on radiation doses to gastrointestinal organs and adrenal tumors has not been analyzed in detail so far. The aim of this study was to quantify the implications of motion during radiation on the dose given to the tumor and adjacent gastrointestinal organs.

## 1. Introduction

Adrenal metastases occur frequently in solid tumors such as lung cancer and melanoma [1]. For oligometastatic and oligoprogressive patients, stereotactic ablative body radiotherapy (SBRT or SABR) of metastases can prolong progression-free survival, and may even increase overall survival [2,3,4,5]. In the upper abdomen, breathing motion, variable organ filling, peristalsis and slow drifts cause inter- and intrafractional variation [6,7]. Additionally, gastrointestinal (GI) organs at risk (OARs) next to adrenal metastases (stomach, duodenum, bowel, colon) are particularly sensitive to high maximum doses [1]. Thus, application of ablative doses can be challenging.

The development of online-adaptive MR-guided radiotherapy (MRgRT) offers several technical features to address these challenges: superior soft tissue contrast, online plan adaptation and imaging simultaneously with irradiation enable the mitigation of interfractional anatomical changes and ensure high doses to the gross tumor volume (GTV), as well as adherence to OAR constraints at each fraction [8,9,10,11]. Breathing motion can be eliminated by gating [12,13]. With adaptive MRgRT accounting for interfractional changes, intrafractional variations still remain as an origin for uncertainties and possibly local failure, as well as OAR toxicities [7]. Both online adaptation and gating prolong fractions [14,15,16]. With mean fraction durations of more than half an hour, fraction times in MRgRT are multiplied compared to conventional SBRT [15]. Consequently, quantification of intrafractional variations and their dosimetric consequences for target volumes and OARs is of uttermost importance.

Several studies have addressed intrafractional variations in the upper abdomen, mainly for pancreatic and hepatic SBRT [17,18,19,20,21]. To the best of our knowledge, only two studies have looked at dosimetric consequences of intrafractional variations in adrenal SBRT. Bernchou et al. assessed the effect of abdominal compression in non-gated SBRT on GTV and OAR doses. However, no specific OAR doses were published [22]. A study by our group investigated the impact of intrafractional motion on OAR doses in 20 abdominal cases, five of which were adrenal metastases, without measuring target volume doses and without quantifying the effect specifically for adrenal metastases [23].

Thus, the impact of intrafractional variations on target volumes and specific OAR doses in adrenal SBRT remains unknown, and this was the motivation for the present study.

## 2. Materials and Methods

Patients treated with adrenal MRgRT between October 2020 and February 2023 were included in the present analysis. Gated, online-adaptive MRgRT was performed with a ViewRay MRIdian © 6 megavolt linear accelerator (ViewRay Inc., Denver, CO, USA). Institutional ethics board approval was obtained for all patients (approvals S-627/2019 and S-862/2019).

MRgRT simulation, planning and treatment procedures have been published before [11]. In brief, simulation imaging comprised a 3D TrueFISP sequence in inspiration breath-hold (axial resolution 1.5 × 1.5 mm^2^ and slice thickness 3 mm). A sagittal 2D CINE sequence (4–8 frames per second) was used for gating. A planning computed tomography (CT) acquired in treatment position was used for dose calculation. GTVs were delineated, including all available information from diagnostic imaging. An isotropic margin of 2 mm was added, respecting the borders of non-infiltrated OARs, to create the clinical target volume (CTV). The planning target volume (PTV) margin was 3 mm isotropically. In most cases, a PTV coverage of 95% with 100% of the prescription dose and an inhomogeneous dose distribution with a maximum of 125% were prescribed. Very large metastases were treated with a homogeneous dose and a maximum of 107%. OAR constraints respected international guidelines [24], and were prioritized over PTV coverage. For the kidneys, stricter constraints were often used to facilitate potential future irradiation of further targets nearby, especially spinal bone metastases.

For daily online adaptation, the 3D MRI was rigidly registered to the simulation MRI, focusing on the GTV. Target volumes were transformed rigidly, and OARs deformably. Structures were recontoured within a PTV_expand_, 3 cm axially and 1 cm craniocaudally around the initial PTV [25]. The liver and kidneys were recontoured completely to calculate mean doses. Forward-calculation of the baseline plan on the anatomy of the day resulted in the predicted plan. In the case of insufficient PTV coverage or OAR constraint violation, an adapted plan was created using the same planning objectives and beam parameters. Immediately prior to dose delivery, another (“preRT”) 3D MRI was acquired to check and correct patient positioning. This step was not required by the manufacturer, but was part of the institutional quality assessment. Both initial and preRT MRI were performed with inspiration breath hold.

The GTV, CTV, PTV and all OARs were retrospectively recontoured on preRT MRIs within the PTV_expand_. The liver and kidneys were recontoured completely. By propagation of the adapted/delivered plans to preRT MRIs, preRT dose distributions were created. These were assumed to represent best the doses actually delivered. The target volume and OAR doses in adapted and preRT plans were compared to assess the dosimetric effects of intrafractional anatomical variations. Non-adapted baseline plans were also propagated to the adapted preRT MRIs for comparison to assess the effect of daily online adaptation, taking into account intrafractional motion.

Normal tissue complication probabilities were computed based on the Lyman–Kutcher–Burman model [26,27,28]. Doses were converted to equivalent doses for the reference dose of the respective model [29]:

(1) $$EQD\left(D\;ref\right)=D(\frac{d+\alpha /\beta \;}{Dref+\alpha /\beta })$$

The equivalent uniform dose (EUD) was calculated for all partial volumes V_i_ with dose D_i_, with EQD_D ref_ as dose D [30]:

(2) $$EUD={\left(\sum_{i}D_{i}^{\frac{1}{n}}\frac{V_{i}}{V_{tot}}\right)}^{n}$$

NTCP [31] was computed as:

(3) $$NTCP=\frac{1}{2\pi }\int_{-\infty }^{t}e^{\frac{{-t}^{2}}{2}}dt$$

(4) $$\mathrm{with}\;t=\frac{\left(EUD-{TD}_{50}\right)}{m*{TD}_{50}}$$

The following three models for GI OAR and parameters were used:

- Gastric bleeding model (TD_50_ = 180, n = 0.12, m = 0.49, D ref = 2.0 Gy, α/β = 2.5 Gy) [32];
- Duodenal toxicity ≥ grade 3 model (TD_50_ = 299.1, n = 0.193, m = 0.51, D ref = 2.0 Gy, α/β = 4.0 Gy) [33];
- Duodenal toxicity grades 2–4 model (TD_50_ = 24.6, n = 0.12, m = 0.23, D ref = 25.0 Gy, α/β = 4.0 Gy) [34].

Statistical analysis was performed with GraphPad Prism v10.3.0 (GraphPad Software, Boston, MA, USA). Statistical significance was assessed with the Wilcoxon signed-rank test for paired, not normally distributed data (dose statistics), with a paired *t*-test for paired, normally distributed data (NTCP), and with the Mc-Nemars test for paired, dichotomic data (violation of constraints). *p*-values < 0.05 were considered statistically significant. The magnitude of *p*-values is graphically indicated by stars: * < 0.05, ** < 0.01, *** < 001, **** < 0.0001.

## 3. Results

### 3.1. Patient and Treatment Characteristics

Twenty-three patients were included in the analysis. One patient received simultaneous SBRT for bilateral adrenal metastases. As the left and right PTV_expand_ did not overlap, both sides were analyzed separately, resulting in 24 adrenal metastases. In total, 200 fractions were analyzed. Relevant patient and treatment characteristics are summarized in Table 1. One patient received only five fractions, although six had been prescribed initially. In two fractions, adaptation was not necessary, and the baseline plan was used. In one patient, two fractions had to be applied at a conventional linear accelerator, due to MR-linac maintenance.

### 3.2. Position Corrections and Time Intervals

From MRI_adapt_ to MRI_preRT_, the median shifts (and ranges) were 0.0 cm (−2.4 cm to +2.20 cm) in the anterior–posterior direction, +0.1 cm (−4.0 cm to +1.5 cm) in the lateral direction and −0.1 cm (−7.1 cm to +6.5 cm) in the craniocaudal direction. The median three-dimensional shift was 1.2 cm (range 0.1 cm to 7.2 cm).

In the 14 left adrenal cases, the PTV_expand_ contained the esophagus in 5 cases, the stomach in 12, the duodenum in 1 and the bowel in 11. In the 10 right adrenal cases, the stomach (n = 1), duodenum (n = 9) and bowel (n = 2) lay inside the PTV_expand_. The ipsilateral kidney was always inside the respective PTV_expand_.

The median time from MRI_adapt_ to MRI_preRT_ was 56:22 [minutes:seconds] (range: 31:46–96:12).

### 3.3. Target Volume Coverage

**Figure 1:**
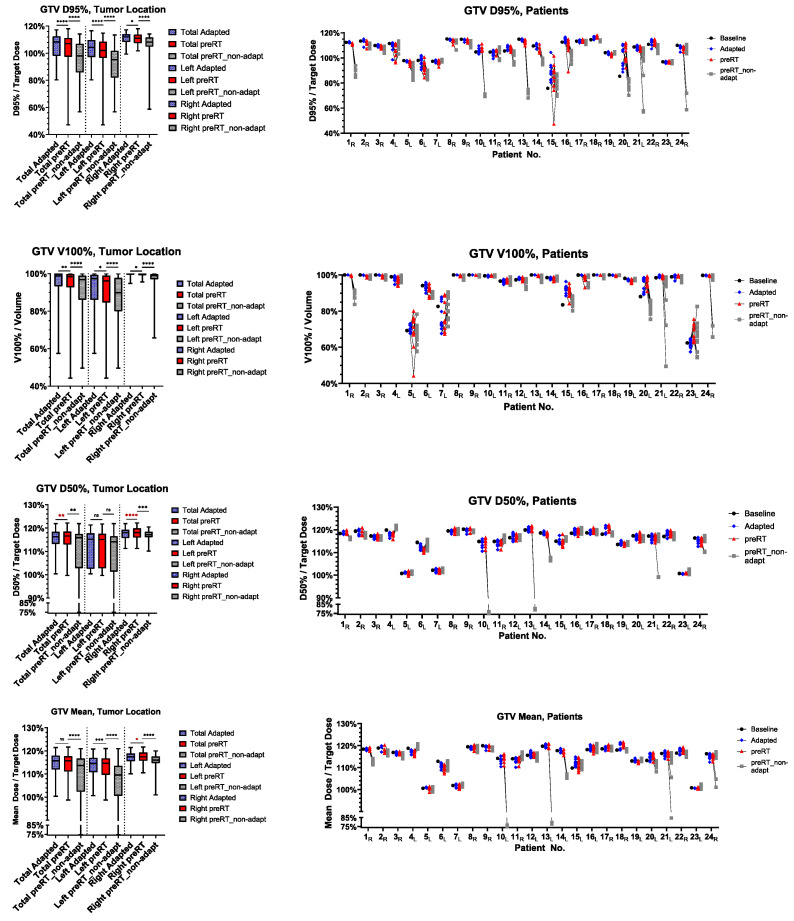
GTV dose metrics of the initial adapted, preRT adapted (“preRT”) and preRT non-adapted plans. Left side: total cohort, left and right adrenal metastases. Right side: fraction-specific dose metrics for individual patients with subscripted characters, indicating the side (L/R). The magnitude of *p*-values is graphically indicated by stars: * < 0.05, ** < 0.01, *** < 001, **** < 0.0001.

**Figure 2:**
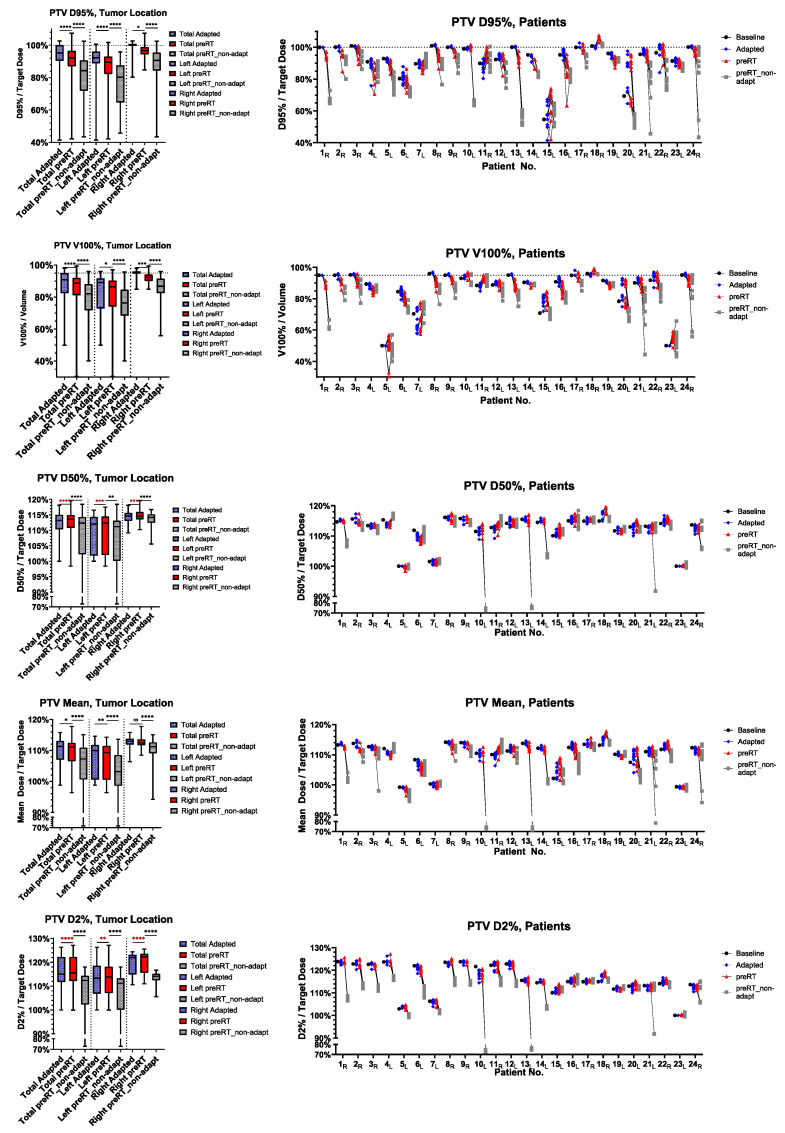
PTV dose metrics of the baseline, initial adapted, preRT adapted (“preRT”) and preRT non-adapted plans. Left side: total cohort, left and right adrenal metastases. Right side: fraction-specific dose metrics for individual patients, with subscripted characters indicating the side (L/R). The magnitude of *p*-values is graphically indicated by stars: * < 0.05, ** < 0.01, *** < 001, **** < 0.0001.

The ratio of preRT plan to adapted plan target volume metrics, such as D95%, V100%, D50%, D2% and D_mean_, are shown in Figure 1 for the GTV and Figure 2 for the PTV. In detail, D95% and V100% were significantly inferior in the preRT plans for GTV and PTV in the total cohort (GTV D95%: *p* < 0.0001, GTV V100%: *p* = 0.0033, PTV D95%: *p* < 0.0001, PTV V100%: *p* < 0.0001) and in left (GTV D95%: *p* < 0.0001, PTV D95%: *p* < 0.0001, PTV V100%: *p* = 0.0260) and right adrenal metastases (GTV D95%: *p* = 0.0377, GTV V100%: *p* = 0.0111, PTV D95%: *p* = 0.0194, PTV V100%: *p* = 0.0003). The GTV D_mean_ was only significantly impaired in left-sided metastases (*p*= 0.0001). GTV D50%, PTV D50% and PTV D2% were not impaired in the preRT plans. Compared to non-adapted plans (“preRT_non-adapt”), the adapted plans (“preRT”) were significantly superior with regard to all target volume metrics in the overall cohort, displayed in Figure 1 and Figure 2 (GTV D50%: *p* = 0.0075, all other metrics *p* < 0.0001, respectively).

### 3.4. Violation of OAR Constraints

**Figure 3:**
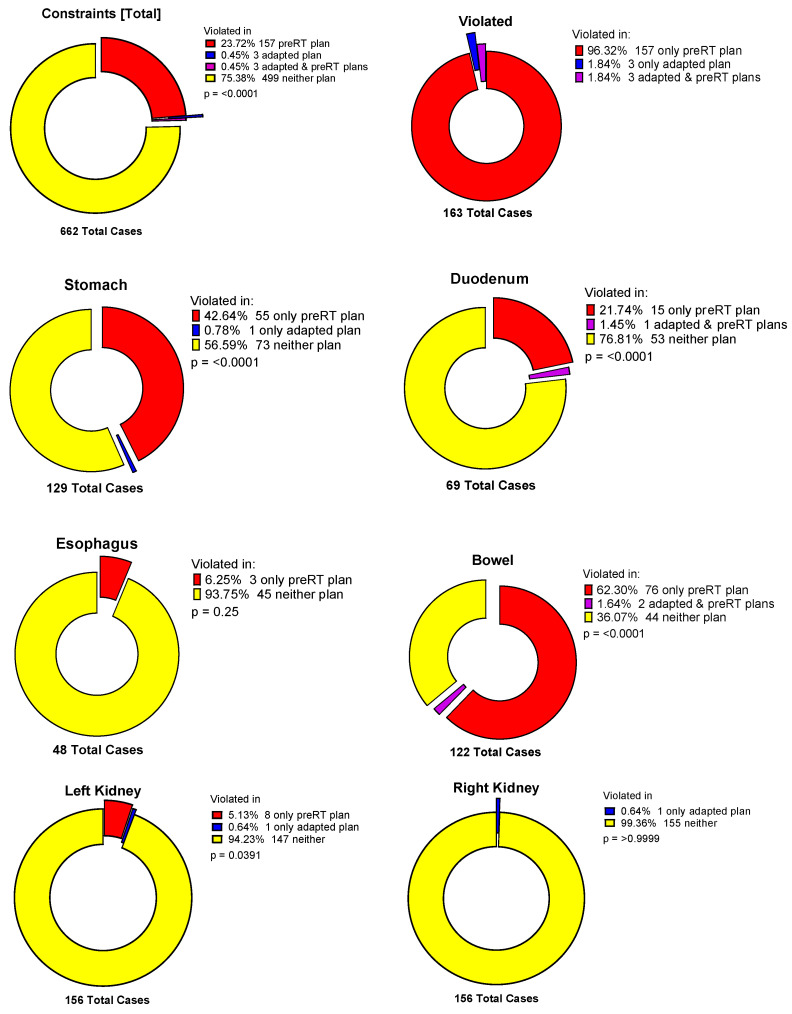
Frequency of constraint violations in total and for specific organs at risk.

Figure 3 illustrates the frequency of OAR constraints violated in adapted and preRT plans. Liver constraints according to international guidelines were never violated. Case-specific kidney constraints were violated in few cases. However, this was always due to the setting of constraints being far stricter than recommended by guidelines, in order to follow the “as low as reasonably achievable” (ALARA) principle.

**Figure 4:**
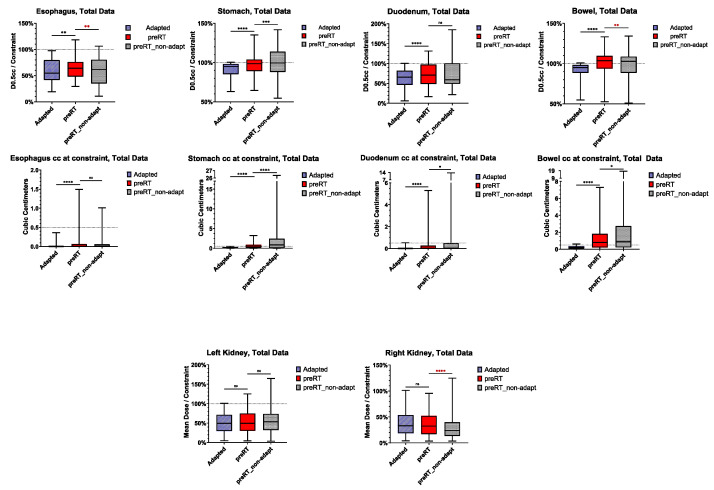
Organs at risk: dose constraints and OAR volume exposed to constrained dose or more in baseline, initial adapted, preRT adapted (“preRT”) and preRT non-adaptive plans. Only patients with overlap of the PTV_expand_ and the respective OAR are included. The magnitude of *p*-values is graphically indicated by stars: * < 0.05, ** < 0.01, *** < 001, **** < 0.0001.

OAR metrics (dose constraints and OAR volume exposed to constrained dose or more) are shown in Figure 4. Individual patient- and fraction-specific metrics are displayed in the Supplement Materials, Figure S1.

**Figure 5:**
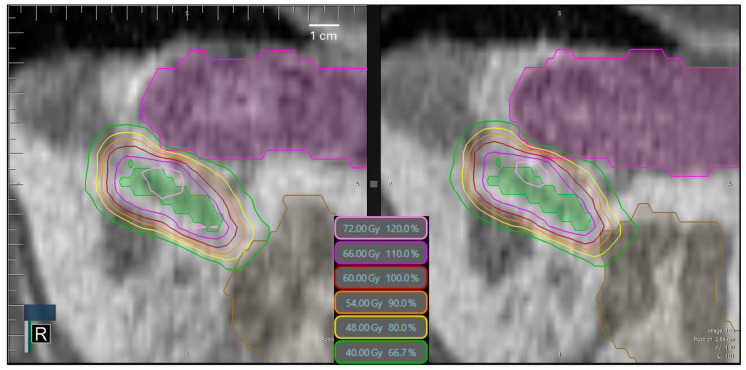
An exemplary case of a left-sided adrenal metastasis (GTV = green ROI) treated in 8 fractions with constraint D0.5cc ≤ 40 Gy for the stomach (purple ROI) and bowel (brown ROI). (**Left**): the adapted and clinically accepted plan. The 40 Gy isodose has only minimal overlap with the stomach and bowel. (**Right**): the preRT plan of the same fraction. The 54 Gy isodose overlaps with both the stomach and duodenum, with a 35% increase compared to the respective constraint.

No significant difference was observed for the kidney mean dose between adapted and preRT plans (left kidney: *p* = 0.7190, right kidney: *p* = 0.9961). However, for all luminal gastrointestinal OARs, the D0.5cc and the volume exposed to the respective dose were significantly higher in the preRT plans (esophagus D0.5cc: *p* = 0.0016, all others: *p* < 0.0001, respectively). Instead of the intended 0.5 cc, the volume exposed to the near-point maximum D0.5cc constraints in single fractions escalated up to 1.5 cc for the esophagus, 3.2 cc for the stomach, 5.3 cc for the duodenum and 7.3 cc for the bowel. Figure 5 illustrates an exemplary case in which both stomach and bowel constraints were violated in the preRT plan. Non-adapted preRT plans would have resulted in a lower median D0.5cc for the esophagus and bowel, but a higher D0.5cc for the stomach. The volumes exposed to the D0.5cc constraint doses were significantly higher for the stomach (*p* < 0.0001), duodenum (*p* = 0.0427) and bowel (*p* = 0.0126). Kidney mean doses would not have been affected negatively in non-adaptive settings (Figure 4).

### 3.5. Normal Tissue Complication Probability (NTCP)

**Figure 6:**
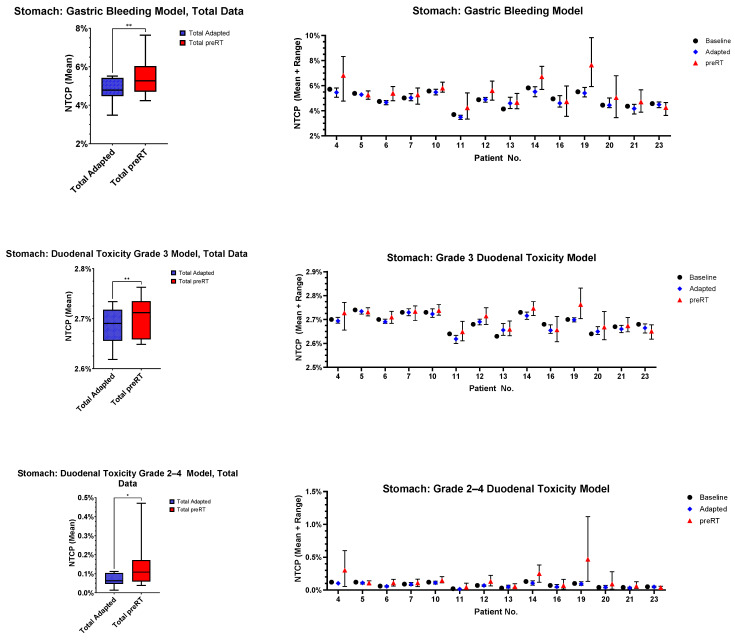

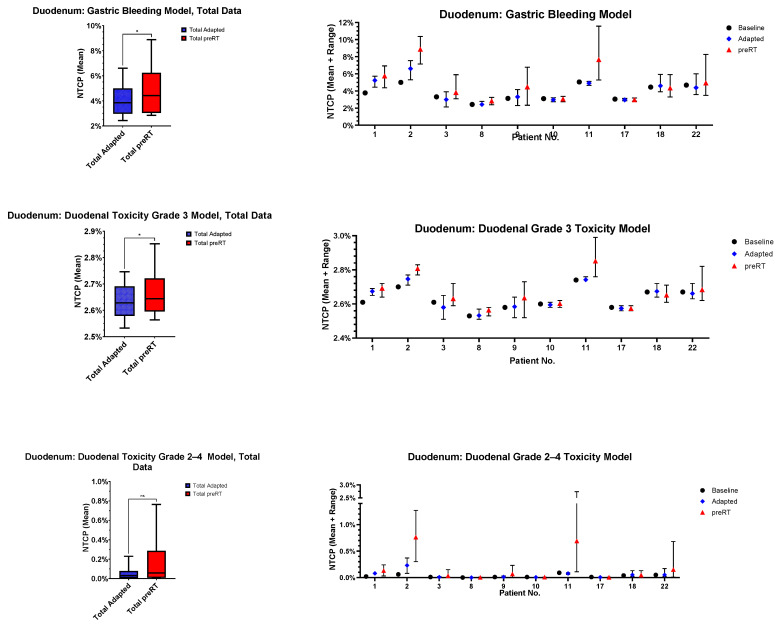

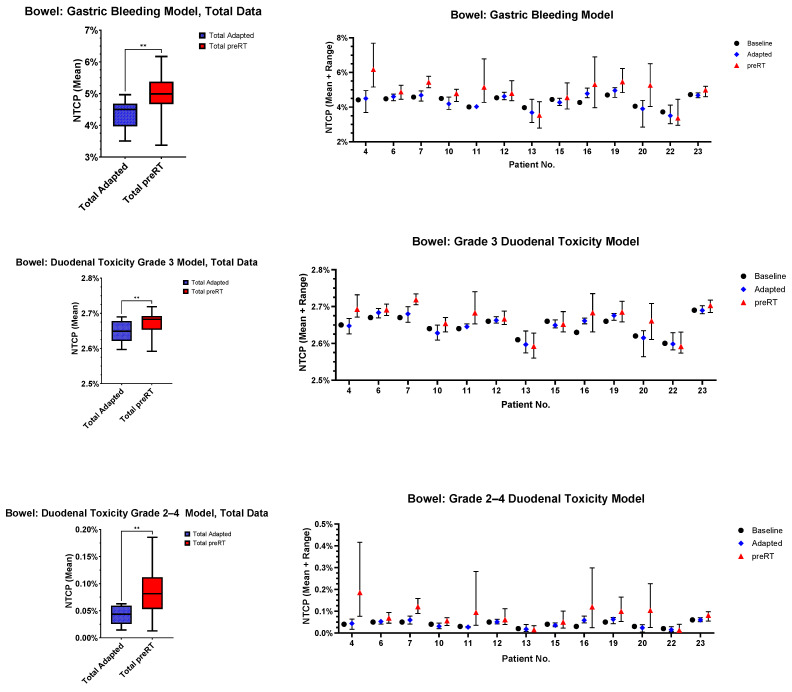
Patient-specific and overall NTCPs for the stomach, duodenum and bowel, according to three different models. NTCPs were calculated as mean values, incorporating all fraction-specific NTCPs. Only patients with overlap of the PTV_expand_ and the respective OARs are included. The magnitude of *p*-values is graphically indicated by stars: * < 0.05, ** < 0.01.

For the OAR with most frequent violation of constraints (stomach, duodenum, bowel), NTCP modeling was performed. The results are depicted in Figure 6. The stomach (*p* = 0.0036, *p* = 0.0055 and *p* = 0.0226) and bowel (*p* = 0.0031, *p* = 0.0038 and *p* = 0.0035) NTCPs were significantly higher in all models, and duodenal toxicity was significantly higher in two out of three models (*p* = 0.0235, *p* = 0.0201 and *p* = 0.0917).

### 3.6. Time-Dependence of PTV Coverage and OAR Overdosage

**Figure 7:**
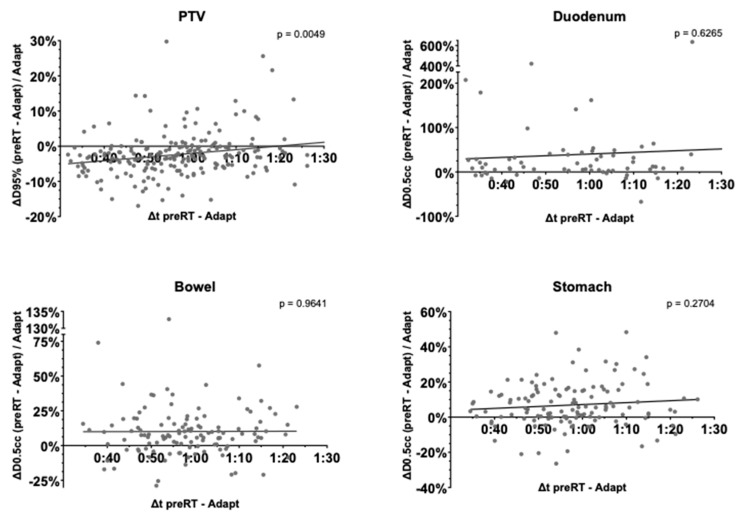
Linear regression to evaluate the dependence of PTV coverage (D95%) and relevant OAR constraints (D0.5cc) in adapted and preRT plans (y-coordinate) on fraction duration (x-coordinate).

Linear regression for the difference in PTV D95% and the stomach, duodenum and bowel D0.5cc between the adapted and preRT plans was computed to assess dependence on fraction duration (Figure 7). Neither gastric (*p* = 0.2704), duodenal (*p* = 0.6265) or bowel (*p* = 0.9641) maximum doses depended significantly on fraction duration. The PTV D95% significantly increased in longer fractions (*p* = 0.0049).

## 4. Discussion

While several studies have assessed intrafractional variability and dosimetric consequences in hepatic or pancreatic radiotherapy, the present work is the first one to address intrafractional changes and effects on target volume coverage and OAR doses specifically and in a homogeneous cohort for adrenal metastases.

Coverage of GTV and PTV, measured as D95% and V100%, was significantly impaired in the preRT plans compared to the clinically accepted, adapted plans. As the dose was prescribed according to these metrics, many of the preRT plans would not have met the approval criteria. A negative impact of reduced GTV and PTV D95% and V100% on local control might be possible. Nevertheless, non-adapted plans calculated based on the preRT MRI anatomy were significantly inferior to adapted preRT plans regarding all relevant GTV and PTV dose metrics. Despite intrafractional motion, online adaption was still highly beneficial for optimizing daily target volume coverage. Prior studies only evaluated the effect on intrafractional motion in non-adaptive SBRT or in adaptive SBRT, without assessing non-adapted baseline plans [17,18,19,20,22,23]. To the best of our knowledge, this work is the first one to explicitly examine the difference in adaptive and non-adaptive plans with regard to dosimetric effects of intrafractional motion in abdominal SBRT. Hence, daily on-table adaptation still highly optimized target volume coverage, and hence, potentially, local control, despite intrafractional motion.

Buergy et al. identified threshold values of GTV D_mean_, GTV D50%, PTV D50% and PTV D2% for local control in adrenal SBRT [35]. Of these metrics, the preRT GTV D_mean_ was significantly lower in left-sided metastases, while the other metrics were not significantly inferior to adapted plans. Previous studies on MRgRT for pancreatic cancer reported no significant reduction in GTV or PTV coverage related to intrafractional motion [17,20].

Violation of OAR constraints occurred frequently in preRT plans. Compared to the 200 plans selected for treatment, additional OAR violations were present in 27% of all fractions for the stomach, 8% for the duodenum and 38% for the bowel. Prior studies on pancreatic MRgRT have reported violation rates of 36–62% for stomach, duodenum and bowel [17,18,20]. In non-adaptive, linac-based pancreatic SBRT, the addition of a 5 mm PRV margin to all gastrointestinal OARs still could not prevent violation of constraints in 20–30% of all cases [19].

Near-maximum doses (D0.5cc) of luminal gastrointestinal organs such as the esophagus, stomach, duodenum and bowel were significantly higher in preRT plans compared to accepted adapted plans. Instead of 0.5 cc, volumes exposed to near-maximum dose constraints were much higher, with up to 3.2 cc for the stomach, 5.3 cc for the duodenum and even 7.3 cc for the bowel, potentially bearing the risk of elevated toxicity. In pancreatic SBRT, comparable maximum volumes (up to 10.9-fold the constraint) exposed to near-maximum dose constraints (here: D1.0cc) have been reported for gastrointestinal OARs [18].

This is also reflected in the NTCP simulations, where gastric, duodenal and bowel NTCPs were significantly elevated in the preRT plans. For example, the simulated median gastric bleeding probability increased from 4.8% to 5.3%. Toxicities reported from clinical trials of adrenal MRgRT were generally low, with maximum grade 3 or higher toxicity rates of 0.9% [36,37,38,39,40]. OAR constraints derived from non-MRgRT data without online adaptation, live imaging and the superior soft tissue contrast of MRI may be stricter than necessary for the level of precision enabled by online-adaptive MRgRT. As kidney constraints were usually stricter than recommended by international consensus guidelines in our study, mild violation of these does not have a clinically relevant impact.

Our results are contradictory to those of Bernchou et al., who concluded that intrafractional motion did not lead to increased violation of gastrointestinal OAR constraints in adrenal MRgRT. However, that finding might be due to the use of abdominal compression in all but one patient [22].

One might assume that intrafractional variations would increase with fraction time, as reported by some authors for thoracic and prostate SBRT [41,42]. However, as linear regression analysis showed, fraction time did not seem to significantly influence gastrointestinal D0.5cc. Upper gastrointestinal intrafractional motion might be dominated by rather random motion, especially peristalsis. PTV D95%, interestingly, even increased with fraction duration. This effect was mainly based on three patients, amongst whom two had the largest GTVs. In these patients with very large metastases, clinical on-table adaptation could not respect the smallest details, which was possible in offline recontouring of the preRT MRIs. Without these three patients, PTV D95% was nondependent on fraction duration. Nevertheless, the time needed for recountering and recalculation of the adapted plans should be kept as low as possible to reduce the risk of relevant anatomical changes. Future implementation of autocontouring and autoplanning into the MR-linac systems will hopefully allow for acceleration of processes. The Ethos system already uses artificial intelligence-supported auto-contouring and autoplanning to reduce the time needed for daily online plan adaptation [43].

The main limitation of the present study is the selection of two time points (linked to MRI acquisitions) to define intrafractional motion. Fully four-dimensional MRI sequences, covering the whole irradiation time, which are not available with the MR-linac, would allow for a more detailed analysis of intrafractional motion [21]. The maximum three-dimensional couch shift between adapted and preRT plans above 1 cm may be larger than intrafractional motion of the GTV or OARs in some cases. However, the most extreme values are most likely linked to rare cases when patients stood up during the adaptation phase because of discomfort or technical problems.

Further, dosimetric analysis and NTCP modeling depend on the underlying contours. In the present study, contouring during actual treatment was performed by three physicians, while for the retrospective evaluation of the preRT plans, all contouring was performed by one observer and controlled by a second one to minimize inter-observer variability. Nevertheless, OAR borders, especially at the transition zones from one GI organ to the other, may vary slightly. NTCP modeling comes with inherent uncertainties, such as the definition of α/β values.

Further limitations of the present work include its retrospective nature, the wide range of patients’ ages and the small sample size of 23 patients, potentially impairing statistical validity. However, with 200 fractions analyzed, the dataset of this work is larger than that of previously published work [22,23].

One method to account for intrafractional OAR motion may be the use of a critical OAR as additional gating structure: the target volume and OAR may be tracked separately. Whenever an OAR overlaps with a certain isodose of the adapted plan of the day (e.g., the maximum point dose tolerated by the respective OAR), the beam is turned off [44]. In the future, online intrafractional replanning may become a valuable option: based on pre-generated motion models and online imaging, deformable vector fields could represent intrafractional target volume and OAR deformation. This could enable online replanning between subsequent beams [45,46].

## 5. Conclusions

Intrafractional motion in adrenal MRgRT caused significant impairment of target volume coverage (D95% and V100%), potentially impairing local tumor control particularly in left-sided metastases. Additionally, frequent violation of gastrointestinal OAR constraints led to elevated normal tissue complication probabilities. In patients with high intrafractional motion, methods like OAR gating could be considered to compensate for these risks. Even in the presence of intrafractional motion, online-adaptive MRgRT significantly improved target volume coverage compared to non-adaptive treatment.

## Figures and Tables

**Table 1:** Patient characteristics. (N)SCLC = (non-)small-cell lung cancer.

Age [y], Median (Range)	63.1 (38.0–81.2)
Sex	
Female	9
Male	14
Primary tumor	
NSCLC	12
SCLC	3
Melanoma	4
Renal cell carcinoma	2
Esophageal cancer	1
Liposarcoma	1
Treatment situation	
Oligometastasis	8
Oligoprogression	15
Laterality	
Left	13
Right	9
Bilateral	1
Fractionation/prescription style	
5 × 10.0 Gy/80% isodose	5
6 × 7.5 Gy/80% isodose	2
8 × 7.5 Gy/80% isodose	3
8 × 5.0 Gy/80% isodose	2
10 × 5.0 Gy/80% isodose	9
12 × 4.0 Gy/homogeneous	3
Frequency of adaptation	
Total fractions prescribed	203
Total fractions treated	202
Adapted	198
Non-adapted	4
Not deemed necessary	2
MR-linac maintenance	2
GTV size [cc], Median (Range)	37.2 (9.9–292.8)

## Data Availability

The data presented in this study will be available upon reasonable request from the corresponding author.

## Supplementary Materials

The following supporting information can be downloaded at https://www.mdpi.com/article/10.3390/cancers17091533/s1: Figure S1: Individual patient- and fraction-specific dose metrics for organs at risk.
