# Supplementary material for: Intrafractional Motion in Online-Adaptive Magnetic Resonance-Guided Radiotherapy of Adrenal Metastases Leads to Reduced Target Volume Coverage and Elevated Organ-at-Risk Doses
Source: Cancers (Basel). 2025 Apr 30;17(9):1533. doi: 10.3390/cancers17091533 (PMC12072169; doi:10.3390/cancers17091533)
Supplement: Supplementary file 1 [file cancers-17-01533-s001.zip › cancers-3534791-supplementary.pdf]

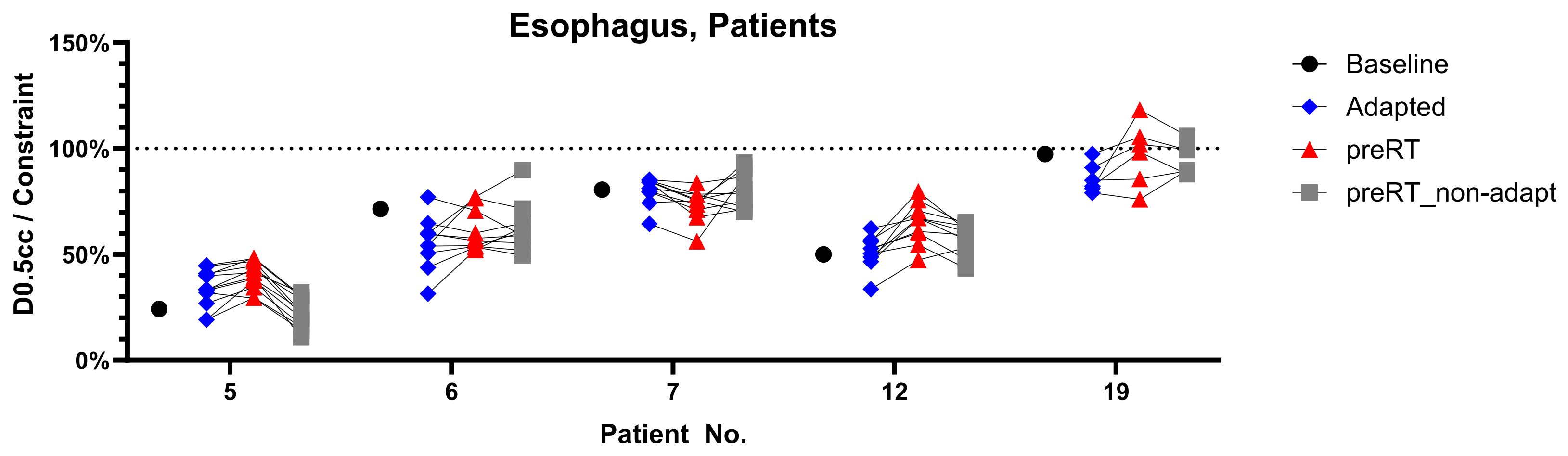
Esophagus, Patients
D0.5cc / Constraint
150%
100%
50%
0%
5
6
7
12
19
Patient No.
Baseline
Adapted
preRT
preRT_non-adapt

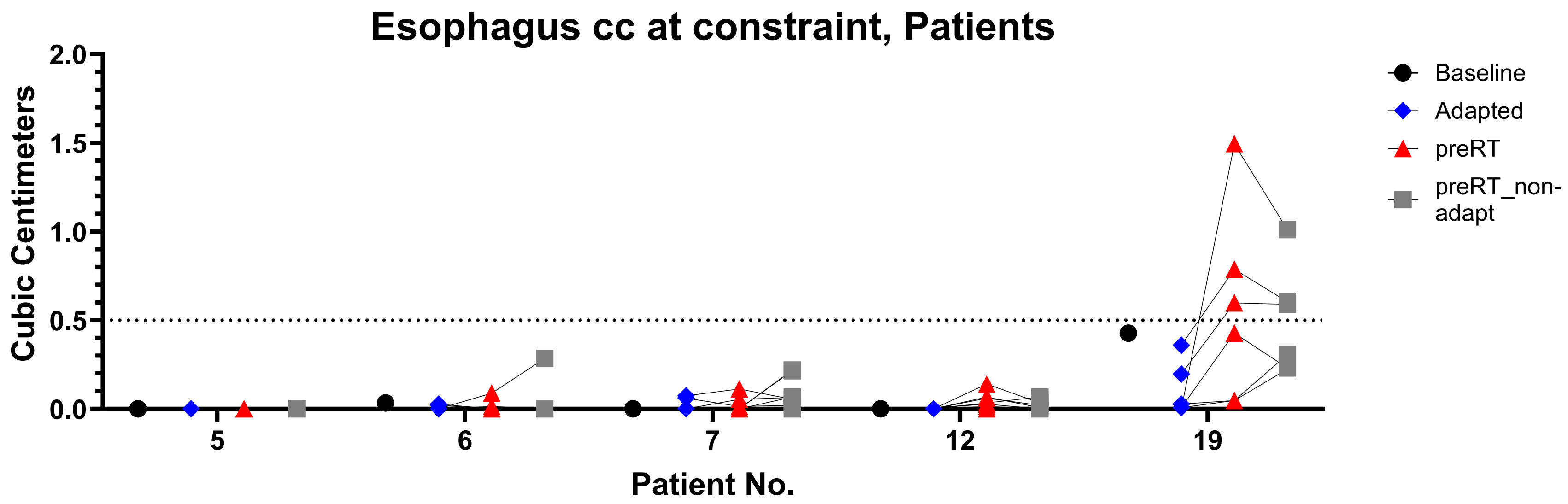
Esophagus cc at constraint, Patients
Cubic Centimeters
2.0
1.5
1.0
0.5
0.0
5
6
7
12
19
Patient No.
Baseline
Adapted
preRT
preRT_non-adapt

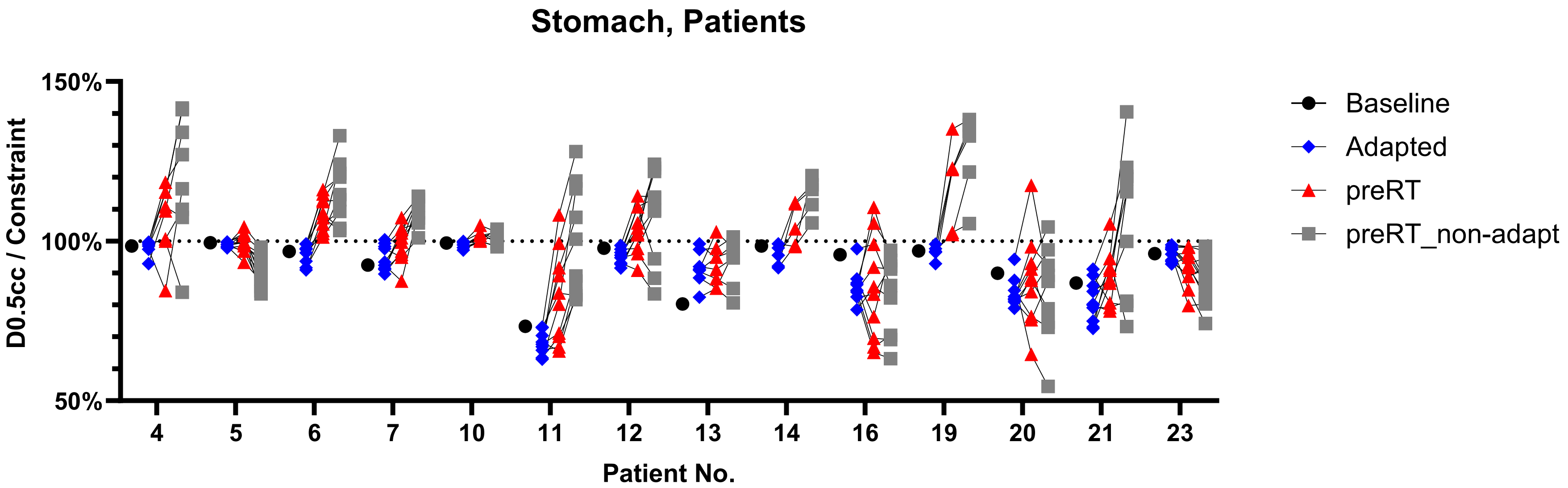
Stomach, Patients
D0.5cc / Constraint
150%
100%
50%
4
5
6
7
10
11
12
13
14
16
19
20
21
23
Patient No.
Baseline
Adapted
preRT
preRT_non-adapt

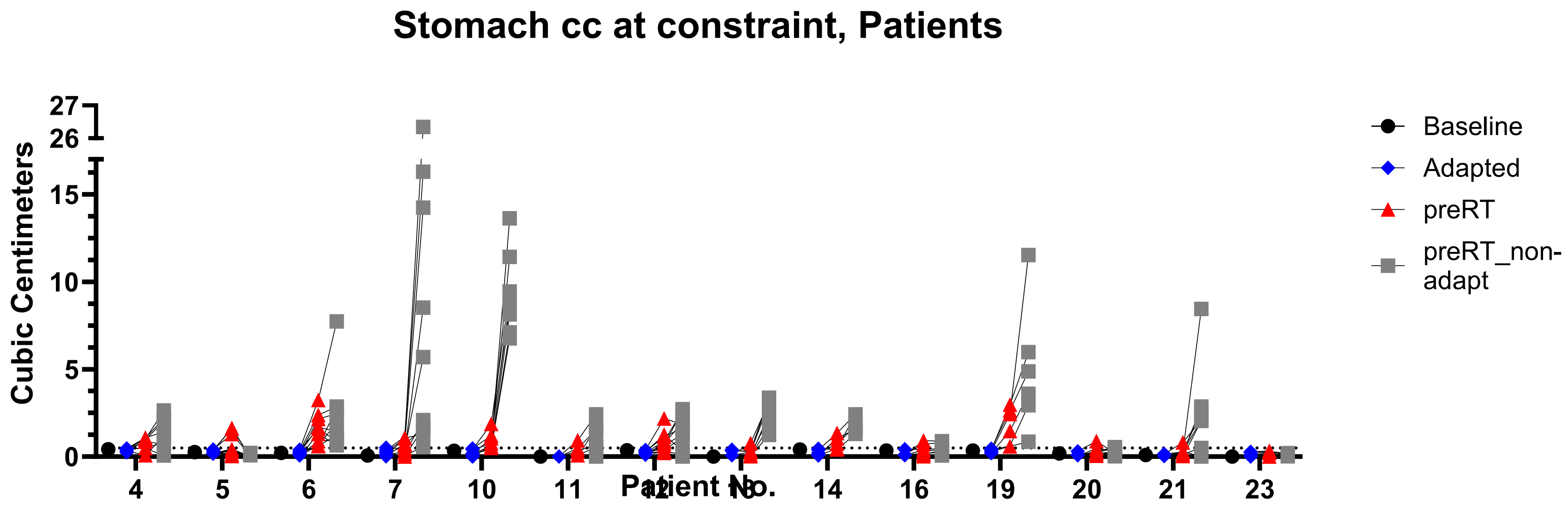
Stomach cc at constraint, Patients
Cubic Centimeters
27
26
15
10
5
0
4
5
6
7
10
11
12
13
14
16
19
20
21
23
Patient No.
Baseline
Adapted
preRT
preRT_non-adapt

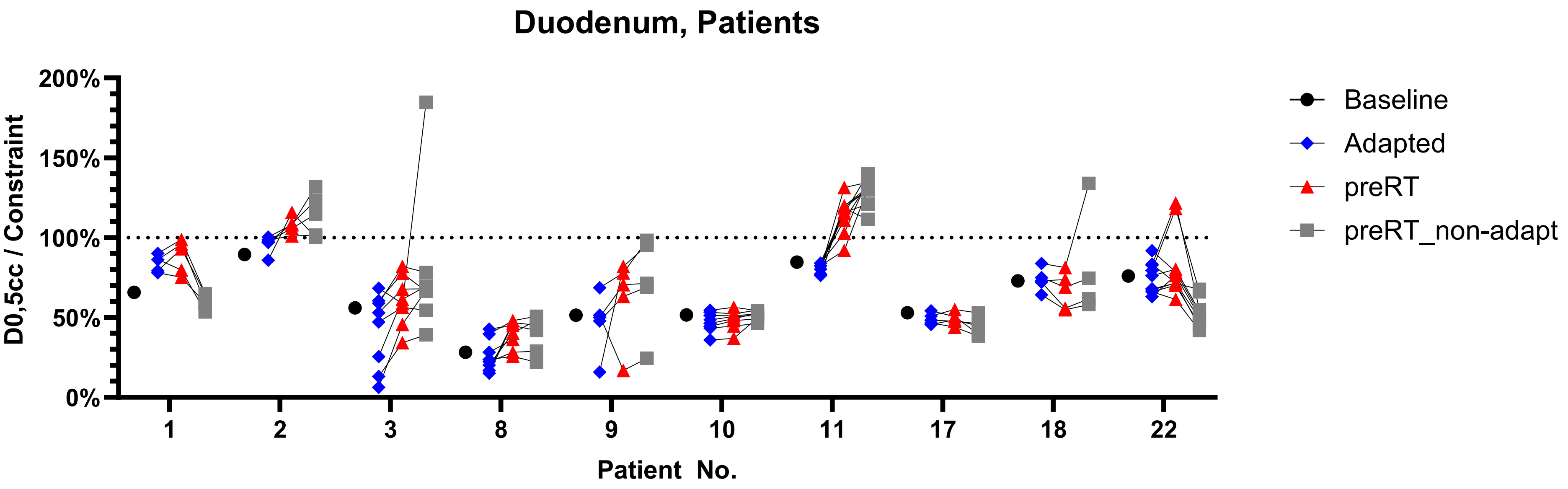
Duodenum, Patients
D0,5cc / Constraint
200%
150%
100%
50%
0%
1
2
3
8
9
10
11
17
18
22
Patient No.
Baseline
Adapted
preRT
preRT_non-adapt

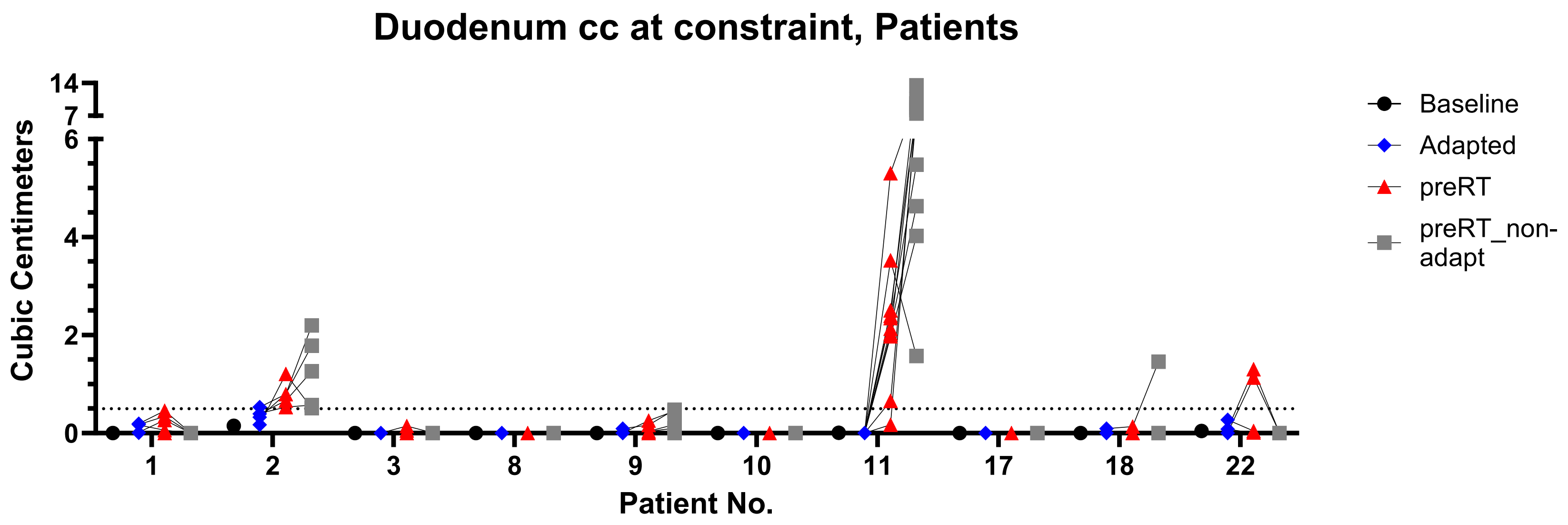
Duodenum cc at constraint, Patients
Cubic Centimeters
14
7
6
4
2
0
1
2
3
8
9
10
11
17
18
22
Patient No.
Baseline
Adapted
preRT
preRT_non-adapt

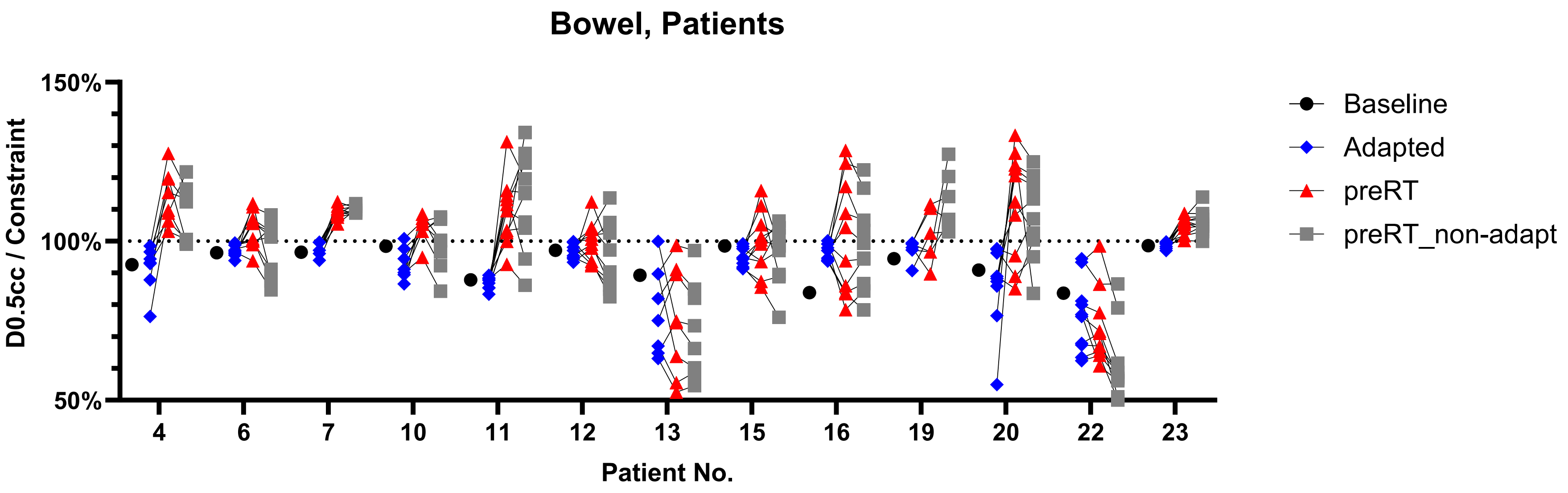
Bowel, Patients
D0.5cc / Constraint
150%
100%
50%
4
6
7
10
11
12
13
15
16
19
20
22
23
Patient No.
Baseline
Adapted
preRT
preRT_non-adapt

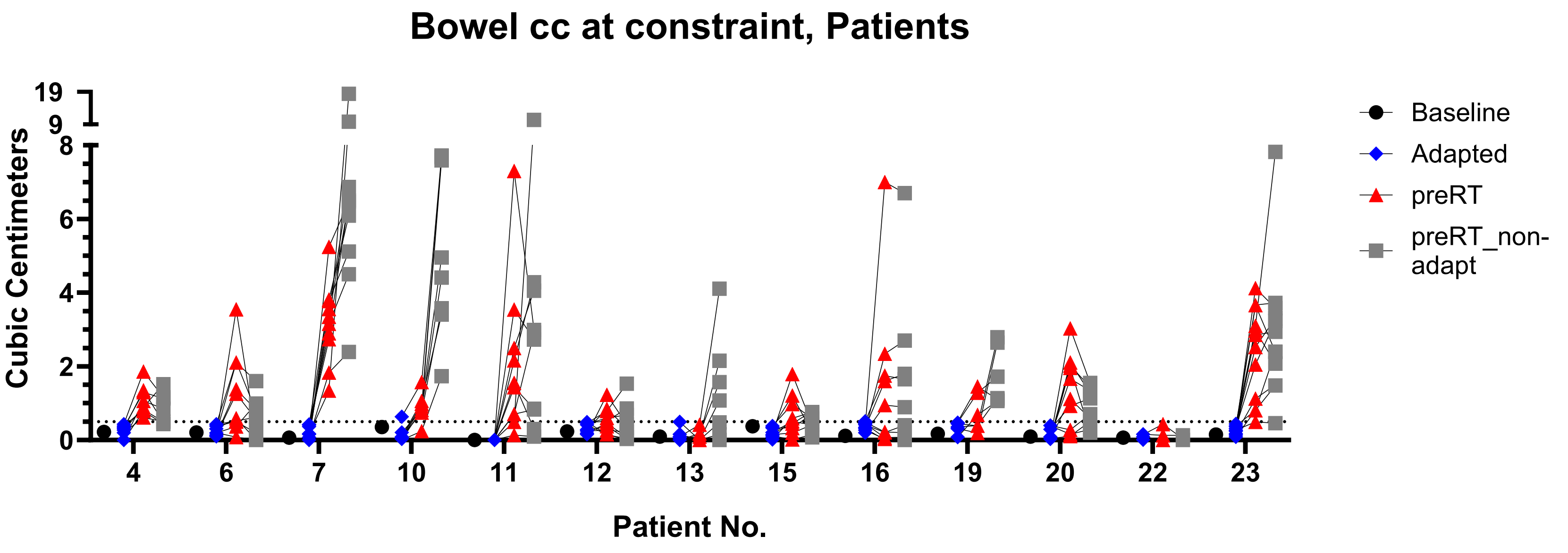
Bowel cc at constraint, Patients
Cubic Centimeters
19
9
8
6
4
2
0
4
6
7
10
11
12
13
15
16
19
20
22
23
Patient No.
Baseline
Adapted
preRT
preRT_non-adapt

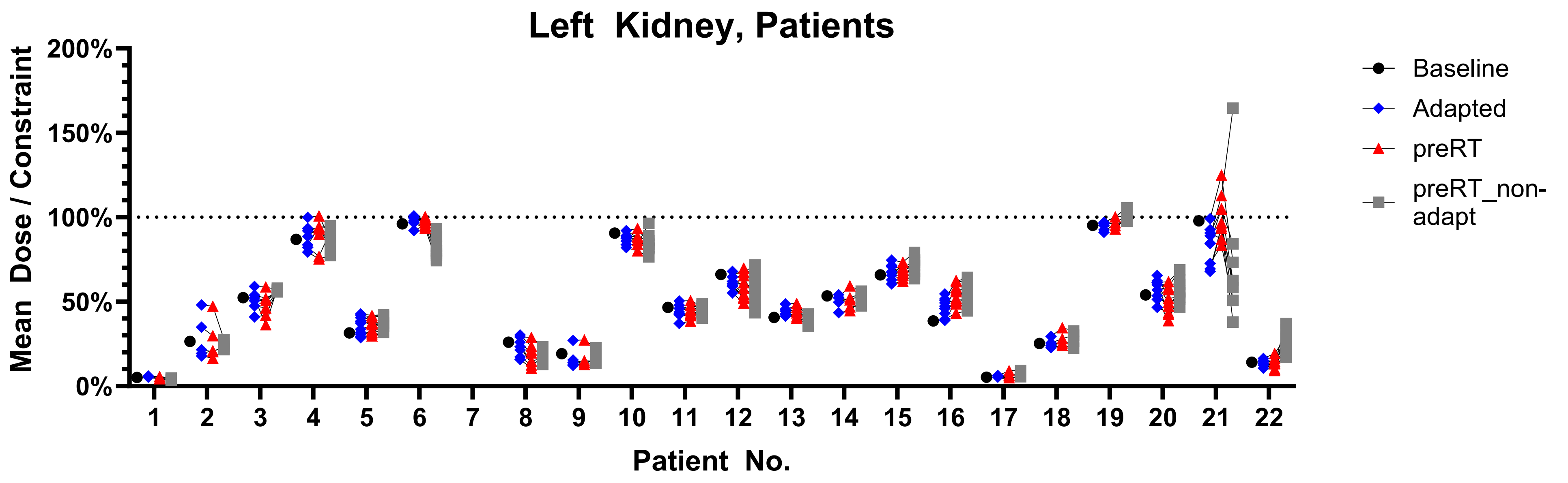
Left Kidney, Patients
Mean Dose / Constraint
200%
150%
100%
50%
0%
1
2
3
4
5
6
7
8
9
10
11
12
13
14
15
16
17
18
19
20
21
22
Patient No.
Baseline
Adapted
preRT
preRT_non-adapt

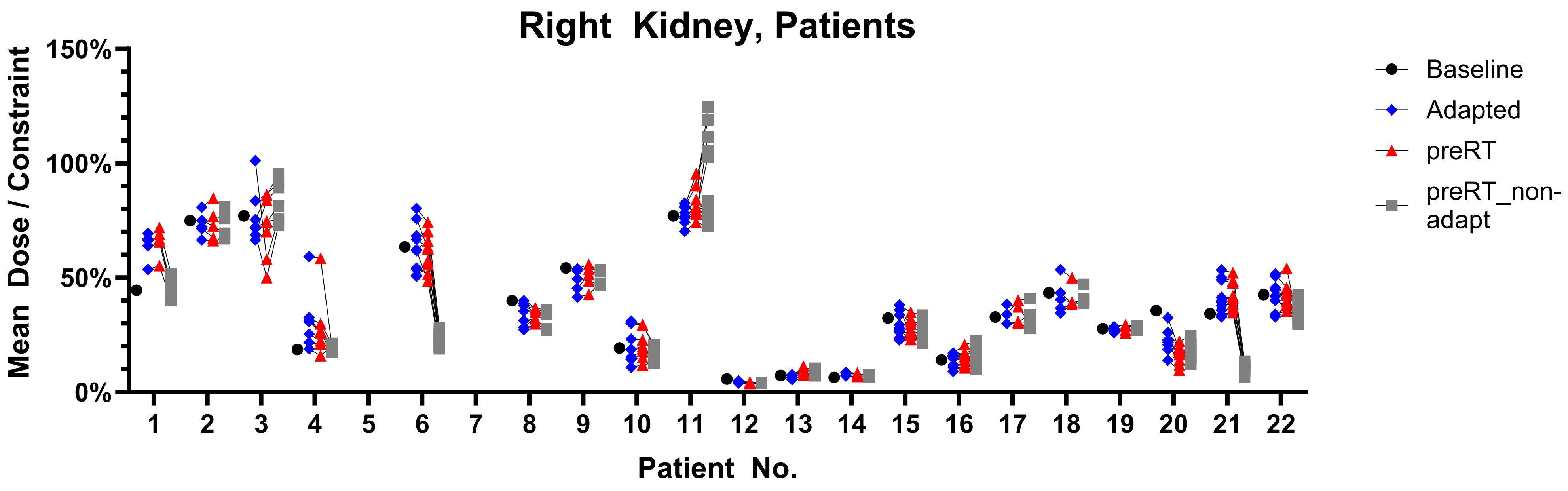
Right Kidney, Patients
Mean Dose / Constraint
150%
100%
50%
0%
1
2
3
4
5
6
7
8
9
10
11
12
13
14
15
16
17
18
19
20
21
22
Patient No.
Baseline
Adapted
preRT
preRT_non-adapt
